# Artificial flowers as a tool for investigating multimodal flower choice in wild insects

**DOI:** 10.1002/ece3.10687

**Published:** 2023-11-20

**Authors:** Kathryn M. Chapman, Freya J. Richardson, Caitlyn Y. Forster, Eliza J. T. Middleton, Thomas E. White, Paul F. Burke, Tanya Latty

**Affiliations:** ^1^ School of Life and Environmental Sciences The University of Sydney Sydney New South Wales Australia; ^2^ Sydney Institute of Agriculture The University of Sydney Sydney New South Wales Australia; ^3^ UTS Business School and Centre for Business Intelligence and Data Analytics University of Technology Sydney Sydney New South Wales Australia

**Keywords:** diptera, flower, flower preference, flower visitors, hymenoptera, multimodal choice

## Abstract

Flowers come in a variety of colours, shapes, sizes and odours. Flowers also differ in the quality and quantity of nutritional reward they provide to entice potential pollinators to visit. Given this diversity, generalist flower‐visiting insects face the considerable challenge of deciding which flowers to feed on and which to ignore. Working with real flowers poses logistical challenges due to correlations between flower traits, maintenance costs and uncontrolled variables. Here, we overcome this challenge by designing multimodal artificial flowers that varied in visual, olfactory and reward attributes. We used artificial flowers to investigate the impact of seven floral attributes (three visual cues, two olfactory cues and two rewarding attributes) on flower visitation and species richness. We investigated how flower attributes influenced two phases of the decision‐making process: the decision to land on a flower, and the decision to feed on a flower. Artificial flowers attracted 890 individual insects representing 15 morphospecies spanning seven arthropod orders. Honeybees were the most common visitors accounting for 46% of visitors. Higher visitation rates were driven by the presence of nectar, the presence of linalool, flower shape and flower colour and was negatively impacted by the presence of citral. Species richness was driven by the presence of nectar, the presence of linalool and flower colour. For hymenopterans, the probability of landing on the artificial flowers was influenced by the presence of nectar or pollen, shape and the presence of citral and/or linalool. The probability of feeding increased when flowers contained nectar. For dipterans, the probability of landing on artificial flowers increased when the flower was yellow and contained linalool. The probability of feeding increased when flowers contained pollen, nectar and linalool. Our results demonstrate the multi‐attribute nature of flower preferences and highlight the usefulness of artificial flowers as tools for studying flower visitation in wild insects.

## Graphical abstract

We used artificial flowers to investigate multi‐attribute flower choice in wild, free‐flying insects.

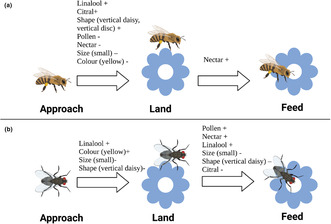

## 1 INTRODUCTION

Flower‐visiting insects pollinate between 65 and 80% of angiosperms, including many economically important crop species (Ollerton et al., 2011) and native plants. Pollination by flower‐visiting insects is thus a key service that structures ecosystems (Potts et al., 2016) and contributes to food security (Aizen et al., 2009; Eilers et al., 2011; Klein et al., 2007).

Generalist flower visitors, which collect pollen from multiple plant families, face the significant challenge of selecting appropriate flowers from a complex foraging landscape composed of co‐flowering plant species. Flower choice is complicated by the fact that flower species can differ dramatically in the concentration, quantity and nutritional composition of the pollen and nectar they provide (Baude et al., 2016; Hicks et al., 2016; Vaudo et al., 2016, 2020). To maximise their fitness, flower visitors may need to consider the perceived value of multiple nutritional attributes; flower choice can therefore be described as a ‘multi‐attribute’ choice problem (reviewed in Latty & Trueblood, 2020).

To further complicate matters, flowers employ a variety of different colours, textures, shapes and odours to attract the attention of foragers. Indeed, flowers have been labelled ‘sensory billboards’ (Raguso, 2004). Flower‐visiting insects may have a hierarchy of floral cue preferences; use different cues at different stages of the decision‐making process; require multiple cues to trigger feeding. For example, the butterfly *Vanessa indica* prefers odourless model flowers in the preferred colour (yellow) over scented model flowers in the non‐preferred colour (Ômura & Honda, 2005), suggesting that the butterflies prioritise colour over scent. In hawkmoths, both olfactory and visual cues are required to trigger feeding responses (Raguso & Willis, 2002). In bees, visual cues are better than olfactory cues at initially attracting bees to artificial model flowers, while olfactory cues are better at promoting landing behaviour (Song et al., 2015). These and other studies suggest that flower choice is a multi‐sensory, multi‐attribute process.

Given the diversity of floral rewards and cues, how do flower‐visiting insects decide which flowers to visit and which to ignore? Traditional optimality models of animal choice, such as optimal foraging theory, assume that animals choose foods that maximise their fitness, subject to cognitive, physiological and informational constraints (Stephens & Krebs, 1986). For example, flower visitors may choose flowers that meet specific nutritional criteria such as high nectar volumes (Bailes et al., 2018; Silva & Dean, 2000) or the presence of specific amino acids (Alm et al., 1990; Broadhead & Raguso, 2021). Foragers are assumed to maximise their fitness by choosing the flowers that provide the highest overall value on attributes of interest.

A number of studies have investigated flower choice by manipulating floral traits on real flowers, for example by altering the colour or pattern of flowers by painting petals (Campbell et al., 2012; Koski & Ashman, 2014) or by clipping petal lobes or removing flowers (Conner & Rush, 1996; Johnson et al., 1995). Manipulative techniques help dissect the impact of single attributes on flower‐visitor behaviour but are often ill‐suited for studying multi‐attribute decision making because it is difficult—and time‐consuming—to manipulate multiple flower traits simultaneously. Physical alteration could also change cues in unwanted ways, such as the release of volatiles or odours after clipping petals or odours from the use of paints. Moreover, flower traits can vary between individuals or populations of the same species (Brink & deWet, 1980; Pleasants & Chaplin, 1983; Real & Rathcke, 1991) and within individuals due to flower position (Lu et al., 2015) or temporal factors (Pleasants, 1983). Flower traits are often correlated (e.g. corolla size and nectar volume) which makes it difficult to disentangle the impact of individual traits.

Artificial flowers can overcome many of the logistical and experimental difficulties of using real flowers in flower choice experiments, as artificial flowers afford control of variation in flower attributes. Artificial flowers have been used to study the impact of flower shape, size and patterning in beeflies (Bombyliidae) (Johnson & Dafni, 1998) and a tabanid fly (Tabanidae) (Jersáková et al., 2012); the impact of olfactory and visual cues on hawkmoth preferences (Raguso & Willis, 2002); preferences for flower symmetry in wild insects (Frey & Bukoski, 2014). It is relatively easy to manipulate a suite of flower traits in artificial flowers, making them ideal tools for investigating multi‐attribute decision making in flower‐visiting insects (for a notable example, see Nordström et al., 2017).

Despite their potential usefulness as tools for understanding decision making in flower‐visiting insects, artificial flowers have only occasionally been used on wild insects. A review of the literature found only six studies (of 160) that used artificial flowers to investigate the behaviour or ecology of flower‐visiting insects outside of enclosed spaces or apiaries (Forster et al., under review). Here, we designed and tested a customisable multi‐attribute artificial flower for use on wild insects. Floral attributes spanned three distinct sensory modalities and included three visual attributes (size, colour, and shape), two olfactory attributes (the odours linalool and citral) and two reward attributes (nectar and pollen substitutes). We used our artificial flowers to answer two key questions:

1. How do flower attributes influence the abundance and diversity of flower‐visiting insects?
2. How do flower attributes affect two phases of the decision‐making process: the decision to land on a flower and the decision to feed on a flower.

## 2 METHODS

### 2.1 Experimental design

Based on preliminary literature searches and our experience, we decided to design our flowers such that they included seven flower attributes: the presence of nectar, the presence of pollen, the presence of citral, the presence of linalool, flower colour, flower shape and flower size. Each attribute had two levels, with the exception of ‘flower shape’ which had four levels. For nectar, pollen, citral and linalool, the levels were simply ‘presence’ or ‘absence’. For shape, the levels were daisy, daisy clustered, simple disc and simple clustered. For colour, the levels were ‘yellow’ or ‘blue’. For ‘display size’, the levels were ‘small’ (5 cm across) and ‘large’ (10 cm across).

**FIGURE 1:**
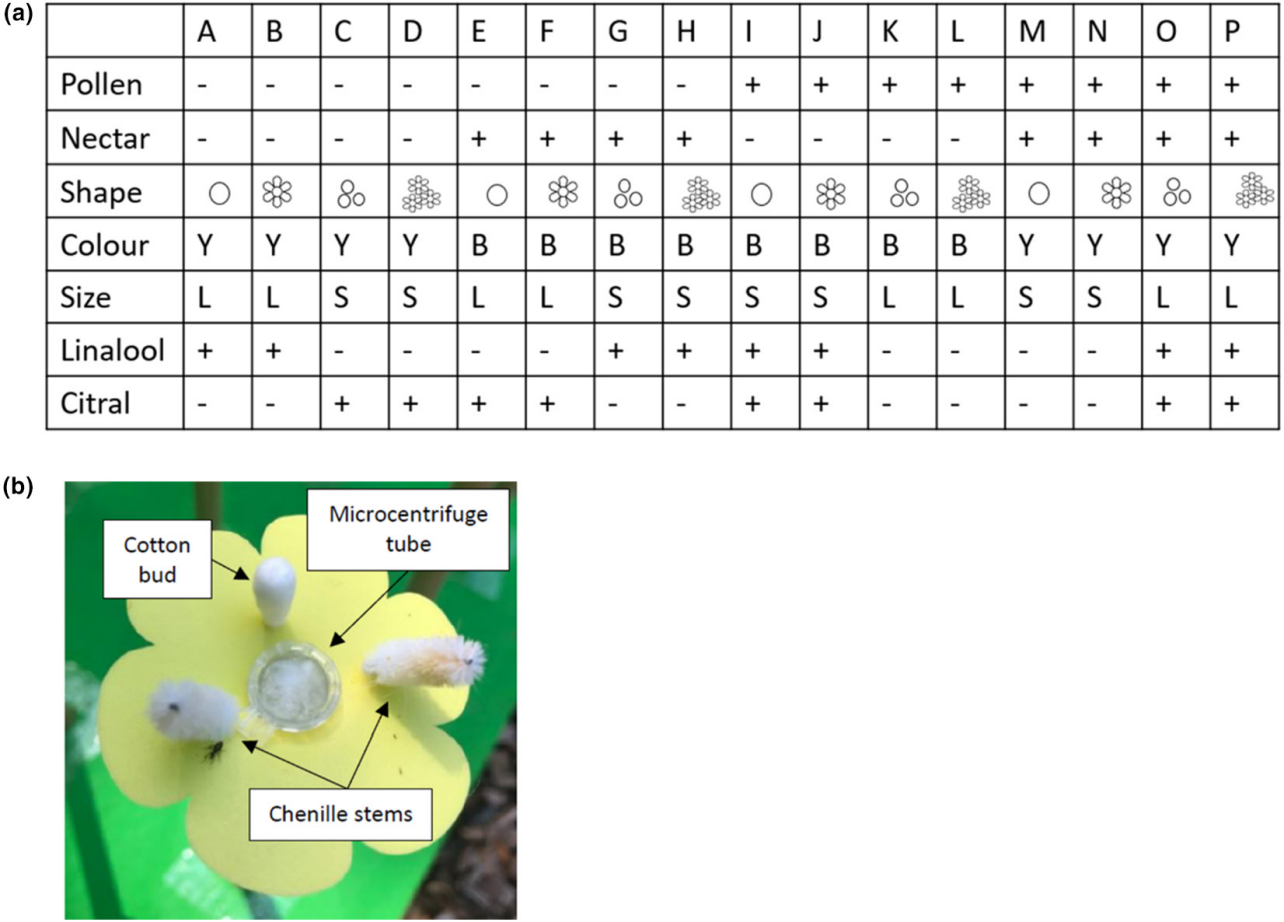
Design of artificial flowers. (a) Shows the combination of traits in each of the 16 flower treatments. For pollen, nectar, linalool and citral, ‘+’ indicates presence, while ‘−’ indicates absence. For colour, ‘Y’ indicates yellow flowers, ‘B’ indicates blue flowers. For size, ‘L’ indicates large flower size and ‘S’ indicates small flower size. The 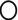 symbol indicates ‘simple disc’, 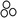 is ‘simple clustered’, 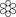 is ‘daisy’ and 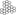 is ‘clustered daisy’ shape. (b) Shows a closeup of an artificial flower including chenille anthers, microcentrifuge tube containing nectar and cotton bud containing odour.

Investigating every combination of our seven attributes in a full‐factorial design was logistically infeasible as it would have required 256 unique flower treatments. To overcome this challenge, we used a fractional factorial experimental design to reduce the number of treatments to a feasible subset of 16 unique flower designs (Figure 1a). Fractional factorial designs are widely used in marketing and engineering in situations where full‐factorial experiments would be cost or time prohibitive (Gunst & Mason, 2009). Fractional factorial designs allow the experimenter to optimally reduce the number of treatment groups while preserving the ability to detect critical effects (Gunst & Mason, 2009). They are limited; however, in that they do not allow for the investigation of interactions between attributes. Nevertheless, the design was appropriate for our experimental aims as we were interested in determining which flower attributes had strong impacts on flower choice rather than on the interactions between attributes (although this is an area of interest for future study).

Various designs are available for two‐level fractional factorial designs for up to 11 factors (Box et al., 1978; Montgomery, 2000). The fractional factorial design we used is an orthogonal main effects design applied to the 2^6^ × 4^1^ full‐factorial design with 256 treatments. By treating the four‐level attribute (flower shape: daisy, daisy clustered, simple disc and simple clustered) as a combination of 2 two‐level attributes (simple disc and simple clustered; daisy and daisy clustered), a resolution four design was employed resulting in 16 treatments thereby allowing each of the main effects between attributes to be estimated using the resulting design (Box et al., 1978; Montgomery, 2000). The design ensures that the correlation between the main effects of the two‐level attributes remain zero, and the correlation between any of the three effects representing the four‐level attribute and any main effect also remain zero. In this regard, the approach avoids issues relating to multicollinearity and biases arising from confounding main effects that would otherwise not be guaranteed relative to using a different design approach (e.g. a subset of treatments based on a completely randomised design). See Appendix S1 for a more detailed explanation of the experimental design procedure.

### 2.2 Flower construction

The corollas of the artificial flowers were made from matte polypropylene display covers (‘Keji Refillable Display Book’) cut to the desired shape using a digital die‐cutting machine (Cricut Explore Air 2; Figure 1b). Each polypropylene ‘flower’ was attached to a 2 ml microcentrifuge tube designed to hold either artificial nectar or plain water. To dispense ‘pollen’, we used 2 cm long chenille sticks as ‘artificial anthers’ (Russell & Papaj, 2016). Chenille anthers were placed on either side of the microcentrifuge tube. Each artificial flower included a cotton bud attached between the two artificial anthers (Figure 1b) which was used to hold odour cues.

Artificial flowers were attached to a ‘stem’ made of 20 mm thick wooden dowel using butyl tape. Each flower was mounted into holes drilled into a bright green square plywood base. Each base held 16 flowers of the same treatment arranged in 4 × 4 rows where flowers were 10 cm apart. To prevent ants and other crawling insects from accessing flowers, we applied a sticky insect barrier called ‘Tanglefoot’ (Tanglefoot Acquisitions, Inc., Grand Rapids, MI, USA) around the holes in the bases.

### 2.3 Flower attributes

#### 2.3.1 Nutritional attributes

We investigated two nutritional attributes: presence/absence of a nectar substitute and presence/absence of a pollen substitute. We initially considered using commercially available honeybee‐collected pollen; however, we were concerned about inadvertently exposing wild insects to honeybee pathogens which can be transmitted via honeybee pollen (Pereira et al., 2019; Schittny et al., 2020). We considered irradiation as a method of sterilising honeybee‐collected pollen; however, some bee pathogens are known to remain infective even after exposure to high levels of gamma radiation (Álvarez Hidalgo et al., 2020; Graystock et al., 2016).

To avoid the risks associated with using honeybee‐collected pollen, we used a commercially available artificial pollen substitute called ‘FeedBee’ (Bee Processing Enterprises Ltd., Toronto, ON, Canada). FeedBee has a nutritional profile comparable to natural pollen and is palatable to honeybees (*Apis mellifera*) (De Jong et al., 2009; Saffari et al., 2010). ‘Feed Bee’ contains 51.8% carbohydrates, 36.4% protein and 3.9% fat (Saffari et al., 2010). We rolled the artificial anthers in FeedBee (herein ‘artificial pollen’) until they were fully coated. In the ‘no pollen’ treatment, artificial anthers were left unbaited.

We used a 45% w/v sucrose solution as an artificial nectar source. In nature, nectar sugar concentrations range between 6 and 85%, with a median concentration of 40% (Pamminger et al., 2019). The sugar concentration in our flowers was therefore within the natural range of nectar concentrations and mimicked an above‐average rewarding flower. To make the ‘nectar present’ flowers, we filled microcentrifuge tubes in the centre of flowers with the sugar syrup; in the ‘nectar absent’ treatments, microcentrifuge tubes were filled with plain water. We placed a small piece of absorbent cotton roll on the top of each tube to prevent smaller insects from drowning in the artificial nectar (Figure 1b).

#### 2.3.2 Visual attributes

**FIGURE 2:**
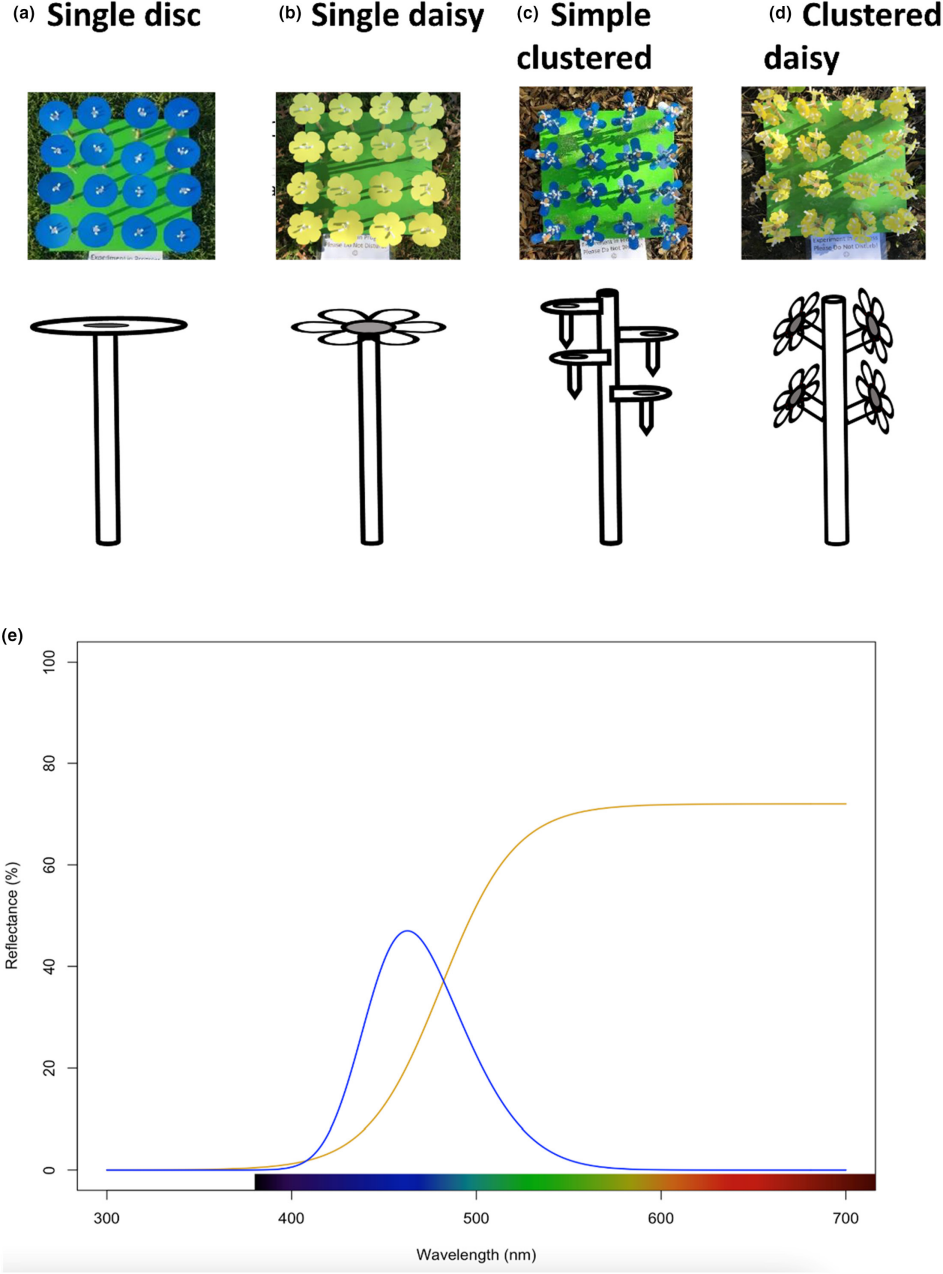
Four flower shapes used in the experiment. (a–d) The top row shows an aerial view of a patch and the picture below shows a schematic of the individual flower. Schematic is not to scale. (e) Smoothed reflectance spectra of the laminated ‘blue’ (blue line) and ‘yellow’ (yellow line) card which we used to construct artificial flowers. Reflectance was recorded using an OceanInsight JAZ spectrometer with PX‐1 pulsed xenon lamp at an integration time of 100 ms, with measurements calibrated against WS‐1 white standard and black velvet dark standard.

We investigated the effect of two flower display sizes (small or large), four shapes (daisy, daisy clustered, simple disc, and simple clustered) and two colours (yellow and blue) (Figure 2). Small flowers measured 5 cm across at the widest point while large flowers measured 10 cm across at the widest point.

The two flower shapes, ‘daisy’ and ‘daisy clustered’, were designed to mimic the general shape of flowers in the Asteraceae (Figure 2). The ‘daisy’ shape was constructed by attaching one daisy‐shaped flower to the top of a dowel. The ‘daisy clustered’ shape was made by sticking five daisy‐shaped flowers into the side of a dowel stem at approximately 45° angles.

The ‘simple disc’ and ‘simple clustered’ flowers were simple geometric shapes not designed to mimic any particular flower. The ‘simple disc’ consisted of a circular disc singly mounted atop a dowel. The ‘simple clustered’ shape consisted of five oval‐shaped discs mounted onto a 23 cm length of dowel at varying levels approximately 2 cm apart in height, starting from the top of the dowel. The dowel was cut to 23 cm (instead of 20 cm) to accommodate the height added by the microcentrifuge tube in the disc shape design; this ensured that all flowers were the same height.

We investigated the impact of flower colour by constructing artificial flower corollas out of matte polypropylene display covers marketed as ‘blue’ or ‘yellow’ (‘Keji Refillable Display Book’) (Figure 2). Reflectance spectra of the yellow and blue flowers were recorded using an OceanInsight JAZ spectrometer with PX‐1 pulsed xenon lamp at an integration time of 100 ms, with measurements calibrated against WS‐1 white standard and black velvet dark standard. Spectra for the two flower colours are presented in Figure 2e.

#### 2.3.3 Olfactory cues

We used two common components of floral odours, linalool (Fluka) and citral (Sigma‐Aldrich Pty Ltd), as odour cues. In both cases, the two levels were ‘presence’ consisting of 1 mL odour cue mixed into 100 mL of water and ‘absence’ consisting of plain water. For treatments that contained both odours, we added 1 mL of each odour to 100 mL of water. We chose a concentration 1 mL/100 mL as this concentration has been demonstrated to attract honeybees (Henning et al., 1992). We applied two drops of either odour solution or water to the cotton bud at the start of the experiments.

### 2.4 Field trials

We deployed patches of artificial flowers around The University of Sydney Camperdown campus in New South Wales, Australia (33.8886°S, 151.1873°E) on the unceded land of the Gadigal people of the Eora nation. Patches were deployed in the morning, between 10:00 and 11:00 am. The study was conducted during Australian autumn (March–May) and early winter (June–August) 2021, when most native bees are dormant (Houston, 2018, p. 69) but when many other insects such as hoverflies (Syrphidae) and honeybees (*A. mellifera*) are abundant and active (Tasker et al., 2020). We tested artificial flowers on days forecast to be sunny with no rain. Maximum temperatures on study days ranged from 18 to 31°C. Experiments ran between 10:00 and 16:00 h.

We placed patches of artificial flowers at 36 pre‐selected experimental sites within The University of Sydney campus. We selected non‐paved lawns or garden beds in low‐foot traffic areas that were not in deep shade. Sites had to be at least 3 m away from flowering plants and at least 10 m away from each other. We considered each patch to be independent as the minimum between‐patch spacing of 10 m was well below the limits of spatial resolution for insect eyes (based on models of the honeybee eye: Hempel de Ibarra et al., 2014); insects would not be able to ‘see’ more than one patch at a time. Moreover, the distance between our patches was 10 times greater than the distance between flowers within a patch, thus creating distinct boundaries between patches. Allocation of patches to sites was randomised and each site was used between 1 and 9 times (median 6 times). We waited at least 7 days before reusing sites. Patch treatment was randomised.

The first observation period began approximately 2.5 h after the patches were deployed. Each flower patch was observed for two 10‐min periods approximately 2 h apart. During the observation period, we recorded the number and identity of all flying insects that came within a 3 cm radius of the artificial flowers. We classified insect–flower interactions as approaches (flew within 3 cm of the flowers without landing), landings (contacted the flower corolla but did not feed) or feedings (mouthparts made contact with liquid in microcentrifuge vial or with the chenille).

Insect visitors were classified to morphospecies on the wing. We did not attempt to capture insects because movements caused some insects to abandon foraging. Consequently, we were limited to relatively coarse identifications for most taxa.

After each observation period, we refilled microcentrifuge tubes with artificial nectar and reapplied two drops of odour (or plain water) to the cotton buds.

All flowers were washed in ethanol at the end of the day to remove any odour cues.

### 2.5 Statistical analysis

Our first goal was to determine whether flower designs differed in their attractiveness to insects. To this end, we used a generalised linear mixed model (GLMM) (glmmTMB, Magnusson et al., 2017) where ‘insect abundance’ (total number of individual insects that landed or fed on flowers) or ‘species richness’ (total number of morphotaxa that landed or fed) was the dependent variable and ‘flower design’ was the independent variable. ‘Flower design’ was coded as a categorical variable with a unique letters assigned as labels for each flower design (see Figure 1). Since we were interested in the general attractiveness of the artificial flowers, we grouped all insects together, regardless of the taxonomic group. We included site ID and day as random effects. Since our data were counts, we used a Poisson distribution with a log link function. We used the package ‘DHARMa’ (Hartig & Hartig, 2017) to visually confirm mixed model assumptions. To determine which treatments differed from one another, we ran pairwise comparisons with the Šidák correction (‘emmeans’ package). A visual examination of plots revealed several points that attracted unusually high numbers of insects and were thus outliers. To determine how these points impacted our results, we re‐ran the analysis with outliers excluded. We defined outliers as values of abundance or species richness that had *z* scores lower than −3 or higher than +3.

We next examined the effect of each flower attribute on total insect abundance (total number of insects that landed or fed on artificial flowers) and species richness (number of morphotaxa that landed or fed on artificial flowers). We used separate GLMMs with ‘insect abundance’ or ‘species richness’ as response variables (package glmmTMB, R statistical Magnusson et al., 2017). Each model included all traits—pollen, nectar, shape, colour, display area, linalool, ciitral—as fixed predictor variables, and site ID, day and observation time as random effects. Since our data were counts, we used a Poisson distribution with a log link function. We used the package ‘DHARMa’ (Hartig & Hartig, 2017) to visually confirm mixed model assumptions. As in the previous analysis, we re‐ran the models excluding outliers that had z scores lower than −3 or higher than +3.

Next, we investigated the effect of flower traits on the type of interaction insects had with our artificial flowers. We divided visits into those where the insect approached (but did not land or feed), landed (but did not feed) or fed (by touching its mouthparts to the liquid in the microcentrifuge containers or to the chenille ‘artificial anthers’). We analysed results for hymenopterans separately from dipterans because these two groups are known to have different preferences for flower traits such as colour, shape and odour, and because we noticed that these two broad taxonomic groups differed in their propensity to approach, land and feed on our artificial flowers. We excluded other arthropod orders (e.g. Orthoptera, Coleoptera, Odonata, Araneae) from this analysis due to low numbers. We used a multinomial regression model (package NNET; Ripley et al., 2016) where the three interaction types (approach, land, feed) were coded as a nominal response variable and the seven flower traits (pollen, nectar, shape, size, colour, linalool, citral) were set as explanatory variables. ‘Approach’ was set as the reference level. We initially included both site and date as additional fixed effects to assess their potential influence, but found their inclusion was not justified (delta‐AIC > 20 in both fly and bee models), and so we subsequently removed them from all models.

All analyses were run in R (version 4.1.2).

## 3 RESULTS

A total of 890 insects interacted with the artificial flowers by approaching, landing or feeding; these represented six insect orders and one arachnid order (Table 1). We further classified visitors into 15 morphotaxa. Two insect orders, Diptera (true flies) and Hymenoptera (bees, wasps, ants and sawflies), made up the vast majority of interactions with artificial flowers. Insects in the order Hymenoptera, consisting primarily of honeybees (*A. mellifera*), were the most common visitors contributing 46.2% of interactions. Insects in the order Diptera, primarily composed of small midge‐like flies, were the second most abundant order, comprising 44.3% of interactions.

**TABLE 1:** Morphotaxa observed on artificial flowers.

Order	Morphotaxa name	Number of interactions
Hymenoptera	*Apis mellifera*	353
Diptera	Diptera small, midge‐like	346
Unknown (likely Diptera)	Unidentified flying insect	52
Hymenoptera	*Tetragonula carbonaria*	37
Diptera	Calliphoridae	25
Diptera	Syrphidae	24
Hymenoptera	Formicidae	13
Lepidoptera	Lepidoptera, unidentified	11
Hymenoptera	Wasp	9
Arachnid	Spider	6
Coleoptera	Coccinellidae	5
Odonata	Damselfly	4
Coleoptera	Coleoptera unidentified	2
Hymenoptera	Unidentified native bee	2
Orthoptera	Grasshopper	1

*Note*: ‘Unknown’ consisted of small, fast flying insects which were very likely Diptera; however, we have listed them as ‘unknown’ as a conclusive ID could not be made on the wing.

Of insects observed feeding on artificial flowers, the majority (87%) consumed artificial nectar. We observed nine instances (2% of feeding events) where insects fed on artificial pollen only and 20 instances where an individual insect consumed pollen and nectar substitutes in the same feeding bout (5% of feeding events). Insect taxa observed feeding on artificial pollen alone included honeybees (*Apis mellifera*; 2 events), stingless bees (*Tetragonula carbonaria*; 2 events), blow flies (*Calliphoridae*; 3 events), an unidentified flying insect (1 event) and a grasshopper (*Orthoptera*, 1 event). Note that the number of pollen feeding events we recorded likely constitutes an under count, as we only counted events where we were certain pollen was being collected (e.g. being actively packed into corbicula) or fed on (mouthparts moving while in clear contact with pollen).

### 3.1 The attractiveness of flower designs

**FIGURE 3:**
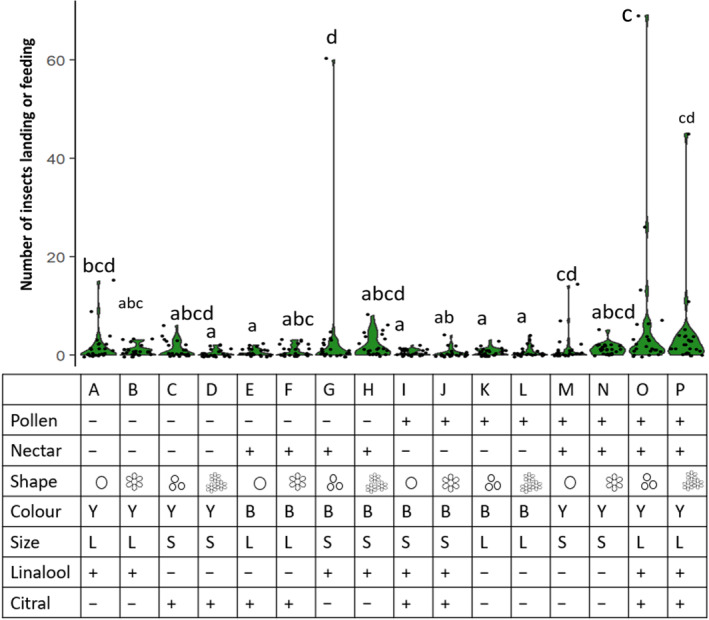
Total number of insects that landed or fed on each of the 16 flower designs. Violin plots sharing a common letter are not significantly different at the .05 level of significance (pairwise comparisons using the Šidák method to correct for multiple comparisons, ‘emmeans’ package, R). The attributes of each flower design are given in the table. For pollen, nectar, linalool and citral ‘+’ indicates presence, while ‘−’ indicates absence. For colour, ‘Y’ indicates yellow flowers, ‘B’ indicates blue flowers. For size, ‘L’ indicates large flower size and ‘S’ indicates small flower size. The 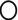 symbol indicates ‘simple disc’, 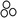 is ‘simple clustered’, 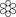 is ‘daisy’ and 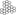 is ‘clustered daisy’ shape.

Flower design significantly impacted the abundance of insects that landed or fed on artificial flowers (Table 2; Figure 3). Of the 16 flower designs, flowers O and P attracted the highest number of landing or feeding insects, followed by flowers G and H. Flowers O and P differed from each other only in shape, such that flower O was the simple clustered shape, while flower P was the clustered daisy shape. Both designs contained artificial nectar and pollen, were yellow, large and contained both linalool and citral (Figure 3). Flowers G and H were also similar to one another but differed in that flower G was a clustered disc shape while flower H was the clustered daisy shape. Both flowers did not contain pollen, contained nectar, were blue, were small, contained linalool and did not contain citral (Figure 3). The qualitative results of the model were not influenced by the removal of 4 outliers which had unusually high levels of visitation. Similarly, the qualitative results did not change when we removed honeybees from the dataset (see Data S1 for details of analysis).

**TABLE 2:** Results of GLMM (Poisson distribution log link function) on the effect of ‘flower design’ on the abundance of insects on artificial flowers.

Predictors	Rate ratio	SE	CI	Statistic	*p*
**Count model**					
(Intercept)	1.65	0.52	0.89–3.06	1.58	.115
Flower design [B]	0.38	0.13	0.19–0.76	−2.73	**.006**
Flower design [C]	0.49	0.14	0.28–0.87	−2.43	**.015**
Flower design [D]	0.17	0.07	0.07–0.40	−4.04	**<.001**
Flower design [E]	0.18	0.07	0.08–0.38	−4.48	**<.001**
Flower design [F]	0.41	0.13	0.21–0.78	−2.72	**.007**
Flower design [G]	1.22	0.39	0.65–2.28	0.62	.537
Flower design [H]	0.68	0.22	0.37–1.26	−1.22	.224
Flower design [I]	0.18	0.07	0.08–0.40	−4.25	**<.001**
Flower design [J]	0.21	0.10	0.09–0.52	−3.41	**.001**
Flower design [K]	0.21	0.08	0.10–0.45	−4.02	**<.001**
Flower design [L]	0.26	0.10	0.12–0.54	−3.58	**<.001**
Flower design [M]	1.10	0.33	0.61–1.99	0.33	.743
Flower design [N]	0.83	0.31	0.41–1.71	−0.50	.615
Flower design [0]	3.03	0.79	1.82–5.04	4.28	**<.001**
Flower design [P]	1.09	0.29	0.64–1.84	0.32	.749

*Note*: Bold text indicates statistical significance at the .05 level. The model included date and site as random effects (results not shown). Marginal *R* ^2^ = .215, conditional *R* ^2^ = .583.

Flower design had a weak impact on species richness, with increased richness on flower O, and decreased richness on flower D (Table 3). The qualitative results of the model were not influenced by the removal of 9 outliers that had unusually high species richness values.

**TABLE 3:** Results of GLMM (Poisson distribution log link function) on the effect of ‘flower design’ on the species richness of morphotaxa on artificial flowers.

Predictors	Rate ratio	SE	CI	Statistic	*p*
Count model					
(intercept)	0.63	0.17	0.38–1.06	−1.73	.083
Flower design [B]	1.00	0.35	0.51–1.98	0.005	.996
Flower design [C]	0.88	0.31	0.43–1.76	−0.37	.710
Flower design [D]	0.41	0.19	0.17–0.99	−1.97	**.048**
Flower design [E]	0.58	0.23	0.26–1.28	−1.35	.177
Flower design [F]	0.64	0.25	0.30–1.38	−1.14	.256
Flower design [G]	0.70	0.27	0.33–1.48	−0.94	.350
Flower design [H]	1.29	0.42	0.68–2.45	0.79	.429
Flower design [I]	0.52	0.22	0.23–1.18	−1.56	.120
Flower design [J]	0.41	0.19	0.17–1.00	−1.96	.050
Flower design [K]	0.76	0.28	0.37–1.58	−0.74	.462
Flower design [L]	0.48	0.21	0.20–1.11	−1.72	.085
Flower design [M]	0.76	0.28	0.37–1.58	−0.73	.467
Flower design [N]	1.30	0.42	0.68–2.46	0.80	.426
Flower design [O]	2.09	0.63	1.16–3.77	2.46	**.014**
Flower design [P]	1.22	0.41	0.63–2.34	0.59	.553

*Note*: Bold text indicates statistical significance at the .05 level. The model included date and site as random effects (results not shown). Marginal *R* ^2^ = .138, conditional *R* ^2^ = .219.

### 3.2 Abundance

The presence of artificial nectar had a significant impact on the abundance of insect visitors such that more insects were observed landing and feeding on flowers that contained a nectar reward (*p* < .001; Table 4). Shape also influenced insect abundance, such that the clustered disc‐shaped flower attracted more insects than the other shapes (Table 4). Yellow flowers were significantly more attractive than blue flowers (Table 4), and insects were more likely to visit artificial flowers that contained linalool (Table 4). The presence of citral decreased the abundance of insect visitors (Table 4). The presence of artificial pollen and flower size did not have a significant impact on insect abundance (Table 4). The qualitative results of the model were not influenced by the removal of four outlier points.

**TABLE 4:** Results of GLMM (Poisson distribution log link function) on the effect of seven floral traits on the abundance of insects on artificial flowers.

Predictors	Rate ratio	SE	CI	Statistic	*p*
**Count model**					
(intercept)	0.23	0.07	0.12–0.43	−4.55	**<.001**
Pollen [y]	1.09	0.15	0.84–1.43	0.64	.523
Nectar [y]	2.69	0.42	1.98–3.64	6.37	**<.001**
Shape [clustered daisy]	0.99	0.21	0.65–1.51	−0.05	.956
Shape [disc]	1.17	0.28	0.73–1.86	0.65	.516
Shape [simple clustered]	2.28	0.42	1.59–3.28	4.47	**<.001**
Colour [yellow]	2.38	0.37	1.75–3.23	5.54	**<.001**
Size [small]	0.93	0.13	0.70–1.23	−0.52	.601
Linalool [y]	1.88	0.25	1.44–2.45	4.65	**<.001**
Citral [y]	0.66	0.10	0.50–0.88	−2.87	**.004**

*Note*: Bold text indicates statistical significance at the .05 level. The model included date and site as random effects (results not shown). Marginal *R* ^2^ = .21, conditional *R* ^2^ = .43.

### 3.3 Species richness

Several flower traits influenced species richness of flower visitors. The presence of nectar or linalool increased the species richness of visitors attracted to artificial flowers (Table 5). Yellow flowers attracted significantly more visits than did blue flowers (Table 5). The presence of citral, presence of artificial pollen, size and shape did not significantly impact insect species richness (Table 5). The qualitative results of the model were not influenced by the removal of nine outlier points.

**TABLE 5:** Results of GLMM (Zero‐inflated Poisson, log link function) on the impact of floral traits on species richness.

Predictors	Rate ratio	SE	CI	Statistic	*p*
**Count Model**					
(Intercept)	0.33	0.08	0.21–0.51	−4.81	**<.001**
Pollen [y]	1.06	0.15	0.81–1.40	0.44	.660
Nectar [y]	1.53	0.22	1.16–2.02	3.01	**.003**
Shape [clustered daisy]	0.98	0.19	0.67–1.44	−0.10	.920
Shape [disc]	0.85	0.17	0.58–1.26	−0.81	.421
Shape [simple clustered]	1.28	0.24	0.89–1.84	1.31	.191
Colour [yellow]	1.58	0.22	1.20–2.08	3.27	**.001**
Size [small]	0.84	0.12	0.64‐ l.ll	−1.23	.218
Linalool [y]	1.32	0.19	1.01–1.74	2.01	**.045**
Citral [y]	0.83	0.12	0.62–1.10	−1.33	.185

*Note*: The model included date and site as random effects (results not shown). Bold text indicates statistical significance at the .05 level.

### 3.4 Interaction type

Hymenoptera and Diptera differed in the relative prevalence of each interaction type. For Hymenoptera, we observed 73 instances (17.7%) where insects approached within 3 cm of artificial flowers but did not land or feed, 16 instances (3.8%) where insects landed on artificial flowers (but did not feed) and 323 instances (78.3%) where individuals approached, landed and fed from the artificial flower. In contrast, we recorded 167 instances (42.2%) where dipterans approached our flowers but did not land or feed, 185 instances (46.9%) where dipterans landed on artificial flowers (but did not feed) and 43 instances (10.8%) where dipterans landed and fed from our artificial flowers.

### 3.5 The impact of flower traits on interaction type

**FIGURE 4:**
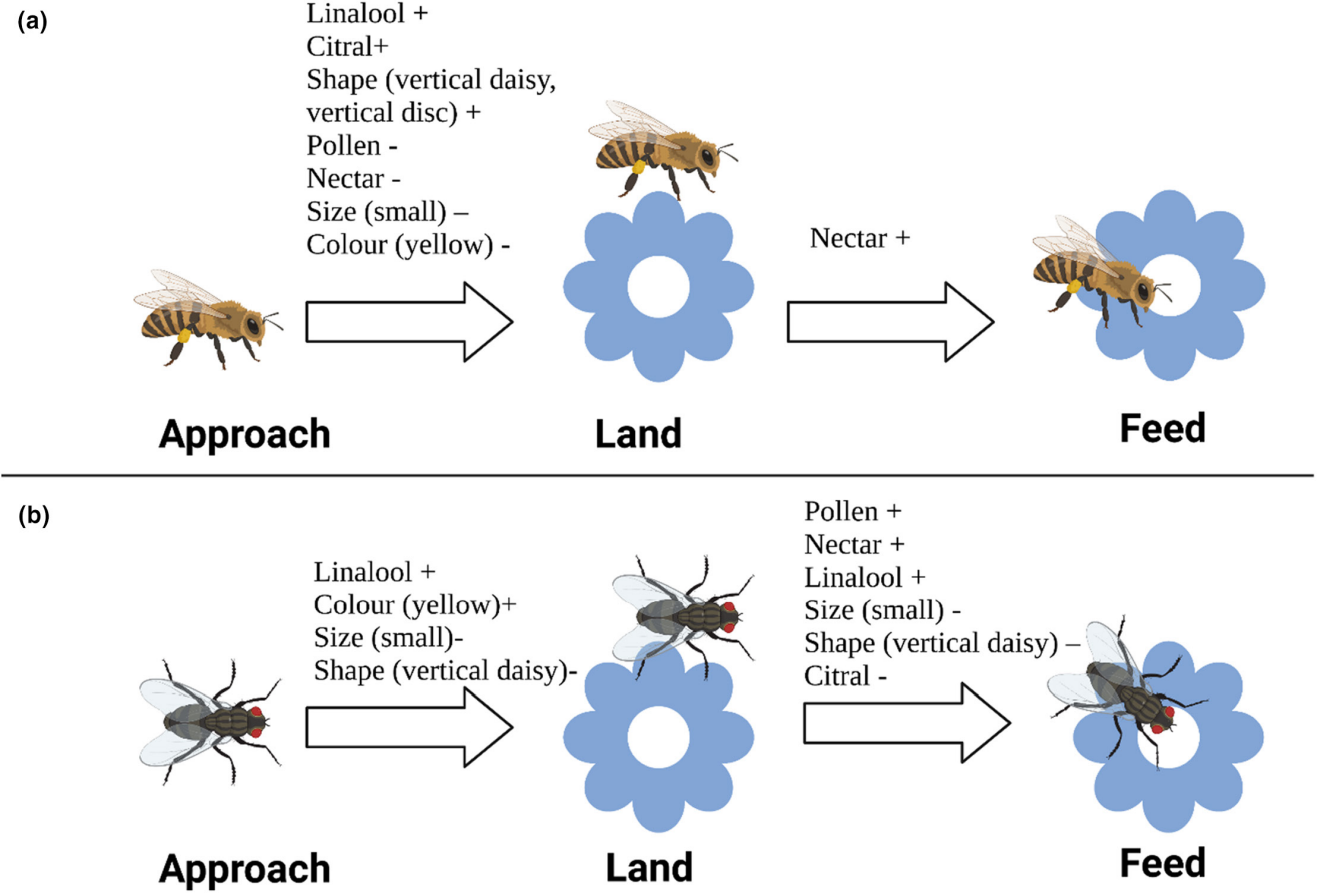
Different traits influence different components of the flower choice decision; (a) hymenopterans (b) dipterans. ‘+’ indicates that a trait increased the probability of a behaviour, relative to approaching; ‘−’ indicates that the trait decreased the probability of a behaviour, relative to approaching. Created with BioRender.com.

For hymenopterans, the probability of landing (but not feeding) on one of the artificial flowers increased (relative to approaching the flower) when the flower was ‘clustered disc’ or ‘clustered daisy’ shape, or contained citral and/or contained linalool (Table 6; Figure 4a). The probability of landing (but not feeding) decreased relative to those that approached (but did not land) when flowers were small, contained nectar, were yellow, or contained pollen. The probability of feeding increased (relative to approaching) when flowers contained nectar (Table 6). None of the other flower traits influenced the probability of feeding (Table 6).

**TABLE 6:** Results of a multinomial regression investigating the effect of floral traits on approaching, landing and feeding by hymenopteran insects.

Predictors	Rate ratio	SE	CI	Statistic	*p*	Response
(Intercept)	0.35	0.25	0.08–1.47	−1.44	.150	Feeding
Pollen [y]	1.86	0.89	0.73–4.75	1.30	.194	Feeding
Colour [yellow]	0.58	0.26	0.23–1.42	−1.20	.232	Feeding
Nectar [y]	8.29	3.81	3.36–20.44	4.61	**<.001**	Feeding
Shape [clustered daisy]	2.16	1.38	0.61–7.57	1.20	.229	Feeding
Shape [disc]	0.60	0.43	0.15–2.42	−0.71	.476	Feeding
Shape [simple clustered]	2.18	1.35	0.64–7.40	1.25	.211	Feeding
Size [small]	1.16	0.55	0.45–2.95	0.31	.758	Feeding
Linalool [y]	1.03	0.47	0.42–2.55	0.07	.941	Feeding
Citral [y]	0.99	0.45	0.40–2.44	−0.02	.982	Feeding
(Intercept)	0.19	0.23	0.02–2.00	−1.38	.167	Landing
Pollen [y]	0.06	0.04	0.02–0.20	−4.74	**<.001**	Landing
Colour [yellow]	0.02	0.01	0.01–0.08	−5.77	**<.001**	Landing
Nectar [y]	0.03	0.02	0.01–0.11	−5.22	**<.001**	Landing
Shape [daisy vert]	30.95	30.11	4.57–209.54	3.53	**<.001**	Landing
Shape [disc]	1.66	2.28	0.11–24.64	0.37	.711	Landing
Shape [disc ve1t]	39.07	35.92	6.41–238.12	3.99	**<.001**	Landing
Size [small]	0.03	0.02	0.01–0.11	−5.52	**<.001**	Landing
Linalool [y]	31.26	22.48	7.60–128.50	4.79	**<.001**	Landing
Citral [y]	26.83	17.60	7.39–97.42	5.02	**<.001**	Landing

*Note*: Bold text indicates significance at the .05 level.

For dipterans, the probability of landing (but not feeding) on one of the artificial flowers increased (relative to approaching) when the flower was yellow and contained linalool (Table 7, Figure 4b). The probability of landing (but not feeding) on one of the artificial flowers decreased (relative to approaching) when the flower was the ‘clustered daisy’ shape and/or was small. The probability of feeding (relative to approaching) increased when flowers contained pollen, contained nectar and/or contained linalool, and decreased when flowers were the ‘clustered daisy’ shape, or contained citral (Table 7, Figure 4b).

**TABLE 7:** Results of a multinomial regression investigating the effect of floral traits on approaching, landing and feeding by dipteran insects.

Predictors	Rate ratio	SE	CI	Statistic	*p*	Response
(Intercept)	0.00	0.00	0.00–0.00	−19.79	**<.001**	Feeding
Pollen [y]	2658.80	992.64	1276.05–5539.93	21.12	**<.001**	Feeding
Colour [yellow]	1.37	0.69	0.51–3.67	0.62	.534	Feeding
Nectar [y]	2.97	1.56	1.06–8.34	2.08	**.039**	Feeding
Shape [clustered daisy]	0.37	0.16	0.16–0.88	−2.27	**.024**	Feeding
Shape [disc]	0.65	0.35	0.23–1.87	−0.80	.422	Feeding
Shape [simple clustered]	0.87	0.36	0.39–1.96	−0.33	.745	Feeding
Size [small]	1.37	0.48	0.69–2.71	0.89	.374	Feeding
Linalool [y]	2238.82	839.96	1070.61–4681.74	20.56	**<.001**	Feeding
Citral [y]	0.00	0.00	0.00–0.00	−15.16	**<.001**	Feeding
(Intercept)	1.57	0.62	0.72–3.41	1.13	.258	Landing
Pollen [y]	0.64	0.16	0.40–1.04	−1.81	.072	Landing
Colour [yellow]	2.77	0.68	1.71–4.48	4.16	**<.001**	Landing
Nectar [y]	0.96	0.23	0.59–1.54	−0.19	.851	Landing
Shape [daisy vert]	0.32	0.11	0.16–0.64	−3.23	**.001**	Landing
Shape [disc]	0.62	0.22	0.31–1.24	−1.35	.177	Landing
Shape [disc vert]	0.52	0.18	0.26–1.04	−1.85	.065	Landing
Size [small]	0.47	0.11	0.29–0.76	−3.12	**.002**	Landing
Linalool [y]	1.78	0.43	I. IO‐ 2.87	2.37	**.018**	Landing
Citral [y]	0.90	0.22	0.55–1.45	−0.44	.657	Landing

*Note*: Bold text indicates significance at the .05 level.

## 4 DISCUSSION

Our multi‐attribute artificial flowers successfully attracted a variety of flower‐visiting insects, including key pollinating taxa such as native bees, honeybees and hoverflies. The attraction of social bees (e.g. *Apis mellifera* and *Tetragonula carbonaria*) to our artificial flowers is particularly encouraging as bees usually require pre‐training in order to recognise artificial flowers as food sources (Ladurner et al., 2005; Rivest et al., 2017). We suggest that artificial flowers offer a replicable, controllable and scalable way to study decision making by wild flower‐visiting insects.

The presence of multimodal cues combined with the presence of food rewards might have encouraged insects to visit the artificial flowers. Previous studies have found that artificial flowers containing both odour and colour cues attract more insects than either cue alone (Kunze & Gumbert, 2001; Milet‐Pinheiro et al., 2012). Although our experimental design does not allow us to test for interactions between traits, it is notable that the two most attractive flower designs contained both rewards (artificial pollen and artificial nectar) and both odour cues (linalool and citral). We suggest that multiple cues improve the attractiveness of artificial flowers and recommend that researchers interested in attracting wild insects construct flowers that contain (at a minimum) odour and visual cues and—where experimental objectives permit—a reward.

Insect abundance and species richness were positively influenced by artificial nectar, which was composed of sucrose and water. Real nectar contains mixtures of sucrose, glucose or fructose (Brandenburg et al., 2009) and amino acids (Baker & Baker, 1977). Since insects may have preferences for nectar with particular sugar compositions (Bachman & Waller, 1977; Broadhead & Raguso, 2021; Erhardt, 1992; Kelber, 2003; Wykes, 1952) or amino acid profiles (Alm et al., 1990; Broadhead & Raguso, 2021; Rathman et al., 1990), our flowers might have excluded species for whom our particular sugar concentration/blend was unappealing. Nevertheless, the fact that we attracted 15 morphotaxa spanning 7 arthropod orders is very encouraging and suggests that sucrose syrups may be sufficient for attracting a range of insect species. Future work could use artificial flowers to investigate the impact of different artificial nectar compositions on abundance, species richness and insect assemblages in wild insects.

The two odorants, linalool and citral, had opposing effects on insect abundance and species richness. The presence of linalool increased insect abundance and species richness, while the presence of citral decreased insect abundance. Linalool is a ubiquitous component of floral odour bouquets thought to serve multiple biological functions, including attracting pollinators (reviewed in Raguso, 2016) and stimulating behavioural responses in house flies, honeybees, fungus gnats and moths (Laloi et al., 2000; Zito et al., 2013; reviewed in Raguso, 2016). Citral is also a component of flower odour bouquets (e.g. Azam et al., 2013; Buchbauer et al., 1993; Martin et al., 2017) and is known to attract honeybees when used alone or in combination with other odorants (Free et al., 1981; Pashte & Shylesha, 2013; Waller, 1970). However, some studies have found citral to be repellent (Giatropoulos et al., 2012), insectididal (Kumar et al., 2013) or an oviposition suppressant (Eben et al., 2020) to dipterans. Insect response to citral is likely concentration‐dependent; for example, (Hao et al., 2013) found that citral is attractive to mosquitos at low concentrations and repellent at high concentrations. It should be noted that the odour concentrations we used here were based on concentrations known to trigger a behavioural response from honeybees (e.g. Henning et al., 1992) and it is possible different species might be attracted/repelled by different odour concentrations. Since the composition and concentration of odour bouquets in real flowers is highly variable, future research would do well to test the influence of a wider range of odours/concentrations alone and when combined in odour bouquets.

Yellow artificial flowers attracted a greater number and diversity of insect visitors than blue artificial flowers. However, our result should be taken with caution as emerging evidence suggests that colour preferences are habitat and species‐specific. For example, (Saunders & Luck, 2013) found that yellow pan traps attracted more native hymenopterans in plant‐diverse orchards, but blue traps were most attractive in native mallee habitats. Similarly, (Nordström et al., 2017) found that hoverflies used different suites of multimodal cues (including colour) in different environments. Species also differ in their colour preferences (Howard et al., 2021; Koethe et al., 2016; Shrestha et al., 2019; Sircom et al., 2018). We suggest that artificial flowers could be a valuable tool for examining variability in flower trait preferences across different landscape types, between different flower‐visiting species and between individuals of the same species.

In addition to insect abundance and species richness, we were also interested in determining how flower attributes influenced the probability that an insect would approach a flower, land on a flower, and feed on the flower. For hymenopterans, the probability of landing on one of the artificial flowers increased (relative to approaching the flower) when the flower was ‘clustered disc’ or ‘clustered daisy shaped’ and contained citral and/or contained linalool. The popularity of the ‘clustered’ flower designs is consistent with a study that found that honeybees were more likely to visit compartments displaying multiple clustered patterns over those displaying a smaller number of patterns (Lehrer et al., 1995). The fact that cues across two sensory modalities (olfaction, visual) increased the probability of hymenopterans landing on artificial flowers reinforces the idea that flower choice is a process that requires multiple cues.

Counterintuitively, pollen and nectar had a negative impact on the probability a hymenopteran would land on the flower (but not feed). Since nectar went on to a have a strong positive impact on feeding, we suggest that the negative impact on ‘landing’ reflected the fact that the presence of nectar tended to increase the probability that hymenopterans would progress to the feeding state (and not be ‘stuck’ in the landing state). Although pollen did not have a statistically significant impact on hymenopteran feeding, we suggest the negative effect of pollen on landing has the same explanation as for nectar. In contrast, dipterans were more likely to progress from landing to feeding if flowers contained pollen and nectar. Most of the dipterans we observed were small‐bodied flies which may have struggled to detect the nectar receptable during approach. This would explain why the presence of nectar had a strong impact on the probability a dipteran would progress from landing to the feeding stage, but no effect on the probability of landing on the flower.

Hymenopterans and dipterans were less likely to land on smaller flowers compared to bigger ones. This preference for larger inflorescences is consistent with previous studies on bees (e.g. Ashman & Stanton, 1991; Galen & Newport, 1987; Martin, 2004). Bigger flowers could increase the total size of the floral display, thus increasing flower detectability. For example, foraging bumblebees (*B. terrestris)* had faster search times when flowers were large compared to when flowers were small (Spaethe et al., 2001). Insects may also use inflorescence size as a proxy for flower rewards, as reward is often positively correlated with metrics of flower size (Armbruster et al., 2005; Ashman & Stanton, 1991; Benitez‐Vieyra et al., 2010; Ornelas et al., 2007; Vandelook et al., 2019).

Hymenopterans were also less likely to land on flowers that were yellow, compared to those that were blue which is unsurprising given the innate preference of several hymenopterans to ‘blue’ hues (Dyer et al., 2016; Giurfa et al., 1995 but see Balamurali et al., 2018 for counter examples). As mentioned above, colour preferences should be interpreted with caution given that colour preference may be driven partially by habitat context. Nevertheless, it is notable that we observed the opposite effect in dipterans, which were more likely to land on yellow flowers (relative to blue).

While multiple factors influenced the decision to land on flowers, only the presence of nectar significantly affected the probability that hymenopterans would feed. Notably, pollen did not significantly affect the decision to feed, even though we observed honeybees and stingless bees actively collecting artificial pollen. Pollen is the primary source of protein and lipids for bees (Wright et al., 2018), so we expected pollen presence to be a key driver of feeding decisions in hymenopterans. Bees may have assessed the pollen substitute as ‘low quality’ and thus were less likely to feed on it; however, research suggests that honeybees consume the pollen substitute we used (FeedBee) at the same rate as natural pollen (Saffari et al., 2010). Nevertheless, we cannot rule out the possibility that insects may have preferences for pollen with particular nutritional features (Ghosh et al., 2020; Russo et al., 2019; Vaudo et al., 2016) that may not have been present in FeedBee. Future experiments are needed to determine the extent to which different insect species accept artificial pollens.

Unlike hymenopterans, the presence of pollen increased the probability that dipterans would begin to feed. However, it is important to note that, unlike honeybees and stingless bees, which collected pollen in their corbicula, it was harder to determine the extent to which dipterans were consuming pollen. Since we defined ‘feeding’ as any contact between mouthparts and the artificial anther, it is possible that dipterans were investigating the pollen‐containing artificial anthers without consuming appreciable amounts. Nevertheless, the presence of artificial pollen promoted interactions with the artificial anthers in a way that appeared consistent with pollen feeding (e.g. insects touching the chenille with mouthparts for prolonged time periods). Closer observation, laboratory feeding trials, or gut dissection would be needed to determine if dipterans did, indeed, consume artificial pollen.

It is possible that our artificial flower design is biased towards attracting some taxa over others. While honeybees made up the majority of flower visitors in our study, we note that multiple studies have found honeybees to be the most abundant flower visitors in the Sydney area (Y. Hanusch & T. Latty, unpublihsed data; McDougall et al., 2022; Tasker et al., 2020), and in Australia more generally (e.g. Nacko et al., 2022; Prendergast & Ollerton, 2021; Tierney et al., 2023; Yates et al., 2005). For example, previous studies in the Sydney region have found that honeybees make up 44% of flower visitors in community gardens (Tasker et al., 2020), 53% of visitors in urban gardens (McDougall et al., 2022) and 85% of visitors in market gardens (Hanusch et al., in preparation). We suggest that the high visitation rates of honeybees to our artificial flowers reflects their high abundance in the environment, although we cannot entirely rule out the possibility that our artificial flowers were particularly attractive to honeybees.

Our results demonstrate the potential role of artificial flowers in studying multi‐attribute choice in flower‐visiting arthropods. Although, for logistical reasons, we restricted each flower attribute to only two levels, future experiments could fix some traits (e.g. colour and odour) while varying the magnitude of other traits. Artificial flowers could then be used to investigate trade‐offs between flower attributes such as nectar concentration and pollen yield, to investigate dose–response relationships in cues such as odour or to examine the interplay between nutritional variables such as amino acid composition and sugar concentration.

Comparisons between the suite of pollinators attracted to artificial flowers versus real flowers in the same floral neighbourhoods would help clarify any biases that may exist. Our work was mainly conducted over Austral winter, when most native bee taxa are inactive, and when there is likely less competition from other flora resources (although it should be noted that Sydney's winter is relatively mild and flowers are present year‐round). Our preliminary work suggests that our artificial flowers are also attractive during the warmer summer months (T. Latty, personal observation), however determining if there are seasonal differences in the attractiveness of artificial flowers and insect preferences will require further testing. Nevertheless, we suggest artificial flowers are an exciting tool for studying a range of multi‐attribute choice behaviours in flower‐visiting animals.

## AUTHOR CONTRIBUTIONS

**Kathryn M. Chapman:** Conceptualization (equal); data curation (equal); formal analysis (equal); investigation (equal); methodology (equal); writing – original draft (equal); writing – review and editing (equal). **Freya J. Richardson:** Conceptualization (equal); data curation (equal); formal analysis (equal); investigation (equal); methodology (equal); visualization (equal); writing – original draft (equal); writing – review and editing (equal). **Eliza J. T. Middleton:** Conceptualization (equal); methodology (equal); project administration (equal); supervision (equal); writing – review and editing (equal). **Caitlyn Y. Forster:** Conceptualization (equal); methodology (equal); supervision (equal); writing – review and editing (equal). **Thomas E. White:** Conceptualization (equal); data curation (equal); formal analysis (equal); methodology (equal); project administration (equal); supervision (equal); visualization (equal); writing – review and editing (equal). **Paul F. Burke:** Conceptualization (equal); formal analysis (equal); methodology (equal); writing – review and editing (equal). **Tanya Latty:** Conceptualization (equal); formal analysis (equal); funding acquisition (lead); methodology (equal); resources (equal); supervision (equal); visualization (equal); writing – original draft (equal); writing – review and editing (equal).

## FUNDING INFORMATION

This work was funded by a grant from the Australian Research Council (DP190101996) to TL.

## CONFLICT OF INTEREST STATEMENT

The authors do not have any conflicts of interest.

## Supporting information


Appendix S1

Data S1

## Data Availability

Data will be archived on Data Dryad after acceptance Dryad doi: https://doi.org/10.5061/dryad.jsxksn0h5. Data files were uploaded as Data S1 with names as follows: Data file ‘freya_kath_data_adj.csv’. Metadata: ‘art_flowers_metadata.xlsx’. R markdown pdf: artificial_flower_2022.pdf.
