# Supplementary material for: Artificial flowers as a tool for investigating multimodal flower choice in wild insects
Source: Ecol Evol. 2023 Nov 20;13(11):e10687. doi: 10.1002/ece3.10687 (PMC10659823; doi:10.1002/ece3.10687)
Supplement: Supplementary file 1 — Appendix S1 [file ECE3-13-e10687-s002.docx]

Appendix 1: Experimental Design

The 16 treatments were based on experimental design theory using a fractional factorial approach. The result is an orthogonal main effects design for a 2^6 x 4^1 experiment in 16 or 2^4 treatments.

The construction proceeds using experimental design based on a suitable aliasing structure summarised as follows:

The full factorial design with 7 attributes, required varying 6 x 2 level attributes and 1 x 4 level attribute. This would require 2^6 x 4^1 = 256 treatments.

The equivalent is to consider the 4-level attribute as a full combination of 2 x two level attributes (i.e., 2^2). This gives rise to an equivalent design of 2^6 x 2^2 = 2^8 = 256 treatments. The advantage of considering attributes as all equivalent two-level attributes, allows a suitable fractional factorial design to be constructed.

Various designs are available for two-level fractional factorial designs for up to 11 factors, originally published in the book *Statistics for Experimenters* by G.E.P. Box, W.G. Hunter, and J.S. Hunter (New York, John Wiley & Sons, 1978) and also given in the book *Design and Analysis of Experiments, 5th edition* by Douglas C. Montgomery (New York, John Wiley & Sons, 2000). We explain the basic principles of fractional factorial design theory for two-level attributes for the present design as follows:

Denoting each of the 8 two-level attributes A to H, the full design requires the main effects of each variable (e.g., A, B, …. ,H), plus higher order interactions such as two-way interactions (e.g., AB, AC, …), three-way interactions (e.g., ABC, ABD, …) and all further higher order interactions to the final eight way interaction (i.e., ABCDEFGH).

The 256 treatments (2^8) design can be reduced to a 2^4 design with 16 treatments using 2^(8-4) design: i.e., 4 main generators.

I = BCDE = ACDF = ABCG = ABDH = ABEF = ADEG = ACEH = BDFG =

BCFH = CDGH = CEFG = DEFH = BEGH = AFGH = ABCDEFGH.

Each main effect, is thereby is NOT confounded in variation with any other main effect (e.g., A) or any other two-way interaction (e.g., AB). This, by definition, is a fractional factorial design of Resolution IV. This does mean each main effect is confounded with a higher order interaction involving three or more variables (e.g., three-way interaction such as ABC, four-way interactions such as ABCD, etc.).

For example, the main effect of A would be confounded with:

A = ABCDE = CDF = BCG = BDH = BEF = DEG = CEH = DFG =

ABCFH = ACDGH = ACEFG = ADEFH = ABEGH = FGH = BCDEFGH.

Hence, the effect of A is not confounded with any other main effect B, C, D, … H nor any of the 28 two-way interactions (e.g., AB, AC, AD, … FG, FG, GH).

It is, however, confounded with seven of the 56 total three-way interactions, namely CDF, BCG, BDH, BEG, CEH, DFG, and FGH. Similarly, it is confounded with three of the 56 total five way interactions, namely, ABCFH, ACDGH, ACEFG, ADEFG, ADEFH and ABEGH. Finally, it is confounded with one of the 8 seven way interactions, namely BCDEFGH.

Likewise, the main effect of H would be confounded with:

H = BCDEH = ACDFH = ABCGH = ABD = ABEFH = ADEGH = ACE = BDFGH =

BCF = CDG = CEFGH = DEF =BEG = AFG = ABCDEFG.

It is noted that the effect of H is not confounded with any other main effect A, B, C, … ,G nor any of the two-way interactions.

The same process can verify the alias structure for all main effects A through H.

The resulting design in 16 treatment is constructed as follows:

| TMT | X1 | X2 | X3 | X4 | X5 | X6 | X7 | X8 |
| --- | --- | --- | --- | --- | --- | --- | --- | --- |
| 1 | -1 | -1 | -1 | 1 | 1 | -1 | -1 | -1 |
| 2 | -1 | -1 | -1 | 1 | 1 | -1 | -1 | 1 |
| 3 | -1 | -1 | -1 | -1 | -1 | 1 | 1 | -1 |
| 4 | -1 | -1 | -1 | -1 | -1 | 1 | 1 | 1 |
| 5 | -1 | 1 | 1 | 1 | -1 | 1 | -1 | -1 |
| 6 | -1 | 1 | 1 | 1 | -1 | 1 | -1 | 1 |
| 7 | -1 | 1 | 1 | -1 | 1 | -1 | 1 | -1 |
| 8 | -1 | 1 | 1 | -1 | 1 | -1 | 1 | 1 |
| 9 | 1 | -1 | 1 | -1 | 1 | 1 | -1 | -1 |
| 10 | 1 | -1 | 1 | -1 | 1 | 1 | -1 | 1 |
| 11 | 1 | -1 | 1 | 1 | -1 | -1 | 1 | -1 |
| 12 | 1 | -1 | 1 | 1 | -1 | -1 | 1 | 1 |
| 13 | 1 | 1 | -1 | -1 | -1 | -1 | -1 | -1 |
| 14 | 1 | 1 | -1 | -1 | -1 | -1 | -1 | 1 |
| 15 | 1 | 1 | -1 | 1 | 1 | 1 | 1 | -1 |
| 16 | 1 | 1 | -1 | 1 | 1 | 1 | 1 | 1 |

First, it can be verified that each level appears an equal number of times across the sixteen conditions (e.g., Attribute X1 has a low level on 8 occasions and high level on 8 occasions).

Second, it can be verified that the correlation matrix of this design is an identity matrix, such that each column is perfectly orthogonal (ie., not confounded) having a correlation of zero. This means in estimation that no main effect is confounded with any other main effect.

Returning to our four-level attribute, the final two columns can be used and thereby gives rise to the following representation:

| TMT | X1 | X2 | X3 | X4 | X5 | X6 | FX78_a | FX78_b | FX78_c |
| --- | --- | --- | --- | --- | --- | --- | --- | --- | --- |
| 1 | -1 | -1 | -1 | 1 | 1 | -1 | -1 | -1 | -1 |
| 2 | -1 | -1 | -1 | 1 | 1 | -1 | 1 | 0 | 0 |
| 3 | -1 | -1 | -1 | -1 | -1 | 1 | 0 | 1 | 0 |
| 4 | -1 | -1 | -1 | -1 | -1 | 1 | 0 | 0 | 1 |
| 5 | -1 | 1 | 1 | 1 | -1 | 1 | -1 | -1 | -1 |
| 6 | -1 | 1 | 1 | 1 | -1 | 1 | 1 | 0 | 0 |
| 7 | -1 | 1 | 1 | -1 | 1 | -1 | 0 | 1 | 0 |
| 8 | -1 | 1 | 1 | -1 | 1 | -1 | 0 | 0 | 1 |
| 9 | 1 | -1 | 1 | -1 | 1 | 1 | -1 | -1 | -1 |
| 10 | 1 | -1 | 1 | -1 | 1 | 1 | 1 | 0 | 0 |
| 11 | 1 | -1 | 1 | 1 | -1 | -1 | 0 | 1 | 0 |
| 12 | 1 | -1 | 1 | 1 | -1 | -1 | 0 | 0 | 1 |
| 13 | 1 | 1 | -1 | -1 | -1 | -1 | -1 | -1 | -1 |
| 14 | 1 | 1 | -1 | -1 | -1 | -1 | 1 | 0 | 0 |
| 15 | 1 | 1 | -1 | 1 | 1 | 1 | 0 | 1 | 0 |
| 16 | 1 | 1 | -1 | 1 | 1 | 1 | 0 | 0 | 1 |

Again, the correlation between the main effects of the two-level attributes remain zero, and the correlation between any of the three effects representing the four-level attribute and any main effect also remain zero.

The design can thereby proceed by replacing the -1s and 1s with the low and high levels for each two-level attribute and replacing the four outcomes corresponding to the four-level attribute.

Of note, is that each attribute appears an equal number of times at each of its levels: For example, attribute A appears eight of the sixteen times at its low level (-1) and eight times at its high level (+1). Similarly, the four-levels of the four-level attribute each appear four times each over the 16 treatments. This implies a balanced design.

The alternative design strategy is to use a RANDOM subset of the full-factorial. This, however, does not ensure that any of the main effects will be uncorrelated with any other main effect, nor occur an equal number of times over the treatments.

Take for example the following random design:

| TMT | X1 | X2 | X3 | X4 | X5 | X6 | X7 | X8 |
| --- | --- | --- | --- | --- | --- | --- | --- | --- |
| 1 | 1 | 1 | 1 | -1 | -1 | -1 | -1 | -1 |
| 2 | -1 | 1 | -1 | -1 | 1 | 1 | 1 | 1 |
| 3 | 1 | 1 | 1 | 1 | 1 | -1 | -1 | -1 |
| 4 | 1 | -1 | -1 | 1 | -1 | 1 | 1 | 1 |
| 5 | -1 | 1 | 1 | -1 | 1 | 1 | 1 | 1 |
| 6 | -1 | 1 | -1 | 1 | -1 | 1 | -1 | -1 |
| 7 | 1 | 1 | 1 | 1 | 1 | -1 | -1 | 1 |
| 8 | 1 | -1 | 1 | -1 | 1 | -1 | 1 | 1 |
| 9 | 1 | -1 | 1 | -1 | -1 | -1 | -1 | -1 |
| 10 | -1 | -1 | 1 | 1 | 1 | 1 | 1 | -1 |
| 11 | 1 | 1 | 1 | 1 | -1 | 1 | 1 | -1 |
| 12 | 1 | -1 | 1 | 1 | -1 | -1 | -1 | 1 |
| 13 | -1 | -1 | 1 | 1 | 1 | -1 | -1 | -1 |
| 14 | -1 | 1 | -1 | -1 | -1 | 1 | 1 | -1 |
| 15 | -1 | -1 | -1 | -1 | 1 | 1 | 1 | 1 |
| 16 | 1 | -1 | 1 | -1 | -1 | -1 | -1 | 1 |
| Appearance of Levels: | | |  |  |  |  |  |  |
| -1 | 7 | 8 | 5 | 8 | 8 | 8 | 8 | 8 |
| 1 | 9 | 8 | 11 | 8 | 8 | 8 | 8 | 8 |

The corresponding correlation matrix is thereby:

|  | X1 | X2 | X3 | X4 | X5 | X6 | X7 | X8 |
| --- | --- | --- | --- | --- | --- | --- | --- | --- |
| X1 |  |  |  |  |  |  |  |  |
| X2 | -0.126 |  |  |  |  |  |  |  |
| X3 | 0.493 | -0.135 |  |  |  |  |  |  |
| X4 | 0.126 | 0.000 | 0.135 |  |  |  |  |  |
| X5 | -0.378 | 0.000 | 0.135 | 0.000 |  |  |  |  |
| X6 | **-0.630** | 0.250 | **-0.674** | 0.000 | 0.000 |  |  |  |
| X7 | -0.378 | 0.000 | -0.405 | -0.250 | 0.250 | **0.750** |  |  |
| X8 | 0.126 | -0.250 | -0.135 | -0.250 | 0.250 | 0.000 | 0.250 |  |

This implies that the correlation between X6 and X7 is extremely high (r=.75), and similarly high amongst attributes X1 and X6 (r=-.63) and X3 and X6 (r=-.674). If the experiment and subsequent model proceeded with such a design, the main effects of attributes 3, 6 and 7 would be subject to high levels of multi-collinearity and therefore bias estimates in the resulting empirical model. A fractional factorial design ensures that the correlation between any two attributes is controlled to be zero and therefore no bias estimates from multicollinearity are presented.

Hence, the experimental approach we have used is a considerable strength of the research methodology.
